# Correction: Wang et al. A VLP-Based Vaccine Displaying HBHA and MTP Antigens of *Mycobacterium tuberculosis* Induces Potentially Protective Immune Responses in *M. tuberculosis* H37Ra Infected Mice. *Vaccines* 2023, *11*, 941

**DOI:** 10.3390/vaccines11091454

**Published:** 2023-09-04

**Authors:** Juan Wang, Tao Xie, Inayat Ullah, Youjun Mi, Xiaoping Li, Yang Gong, Pu He, Yuqi Liu, Fei Li, Jixi Li, Zengjun Lu, Bingdong Zhu

**Affiliations:** 1 Gansu Provincial Key Laboratory of Evidence Based Medicine and Clinical Translation, Lanzhou Center for Tuberculosis Research, Institute of Pathogen Biology, School of Basic Medical Sciences, Lanzhou University, Lanzhou 730000, China; wangj19@lzu.edu.cn (J.W.); xiet18@lzu.edu.cn (T.X.); miyj@lzu.edu.cn (Y.M.); gongy20@lzu.edu.cn (Y.G.); lf@lzu.edu.cn (F.L.); 2 Institute of Pathogenic Physiology, School of Basic Medical Sciences, Lanzhou University, Lanzhou 730000, China; 3 Respiratory Department of Lanzhou Pulmonary Hospital, Lanzhou 730000, China; 4 State Key Laboratory of Genetic Engineering, School of Life Sciences, Fudan University, Shanghai 200438, China; lijixi@fudan.edu.cn; 5 Lanzhou Veterinary Research Institute, Chinese Academy of Agricultural Sciences, Lanzhou 730000, China; 6 State Key Laboratory for Animal Disease Control and Prevention, College of Veterinary Medicine, Lanzhou University, Lanzhou 730000, China

Concerns were brought to the attention of the journal’s editorial office after the paper was published [1]. After conducting an investigation in collaboration with the academic editors, the authors wish to make the following corrections to this paper:

1. We changed the title of the paper from “A VLP-Based Vaccine Displaying HBHA and MTP Antigens of *Mycobacterium tuberculosis* Induces Protective Immune Responses in *M. tuberculosis* H37Ra Infected Mice” to “A VLP-Based Vaccine Displaying HBHA and MTP Antigens of *Mycobacterium tuberculosis* Induces Potentially Protective Immune Responses in *M. tuberculosis* H37Ra Infected Mice”.
2. We added the content “and reduced the bacterial load in the lungs of mice infected with *M. tuberculosis* H37Ra” to the second-to-last sentence in the Abstract, so the sentence should be changed to: “We demonstrated that LV20 combined with the adjuvant composed of DDA and Poly I: C (DP) elicited significantly higher antigen-specific antibodies and CD4+/CD8+ T cell responses than PBS and BCG vaccination in mice, and reduced the bacterial load in the lungs of mice infected with *M. tuberculosis* H37Ra.”
3. We added a second paragraph to Section 2.7:

“Our lab found that prolonging the intervals of immunization could improve the protective efficacy compared to schedule of 0, 21 and 42 days [35]. Therefore, in the evaluation of protective efficacy against *M. tuberculosis* H37Ra, we chose an immunization schedule of 0, 28 and 84 days. C57BL/6 mice were immunized with BCG and PBS onetime inoculation at day 0. HBHA/DP, LV20 and LV20/DP were immunized subcutaneously three times at 0, 28 and 84 days. The protective efficacy was detected following H37Ra (5 × 10^6^ CFU in 50 μL per mouse) intranasal challenge at 84 days after the last immunization. PBS and BCG groups were used as control. The number of mice per group was at least 5.”

In this paragraph, an additional reference was cited and numbered as reference 35; the references hereafter have been re-ordered accordingly:

35. Bai, C.; He, J.; Niu, H.; Hu, L.; Luo, Y.; Liu, X.; Peng, L.; Zhu, B. Prolonged intervals during *Mycobacterium tuberculosis* subunit vaccine boosting contributes to eliciting immunity mediated by central memory-like T cells. *Tuberculosis (Edinb)* **2018**, *110*, 104–111.

4. We added Section 3.6, which includes a new paragraph and the original Figure S2 from the Supplementary Material as Figure 7:

*3.6. Protective Efficacy of LV20/DP against Challenge with M. tuberculosis H37Ra*

Eighty-four days after the last immunization, mice were challenged with *M. tuberculosis* H37Ra 5 × 10^6^ CFU via the intranasal route, and bacterial counts in the lungs were enumerated 21 days later. It was found that the bacterial loads in LV20/DP group reduced approximately 1.18 log10 CFU compared to the HBHA/DP group and reduced approximately 1.3 log10 CFU compared to PBS control (*p* < 0.01; Figure 7), indicating that LV20/DP induced higher protective efficacy than HBHA/DP and PBS groups and similar to that of the BCG group. Interestingly, it was revealed that LV20 without adjuvant did not induce protective immunity against mycobacterial infection.

**Figure 7:**
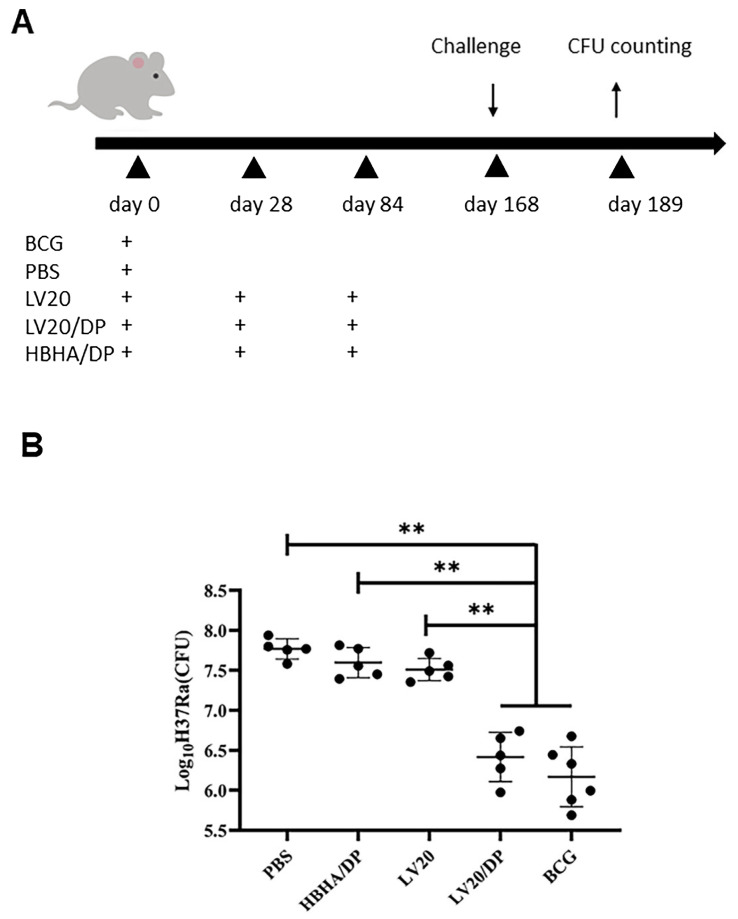
Immunization schedule and protective efficacy of immunization with LV20 in adjuvant DP against *M. tuberculosis* H37Ra infection. (**A**) The immunization schedule to assess protective efficacy. C57BL/6 mice were immunized with BCG and PBS one-time inoculation on day 0. HBHA/DP, LV20 and LV20/DP were immunized subcutaneously three times on days 0, 28 and 84. H37Ra (5 × 10^6^ CFU in 50 μL per mouse) intranasal challenge occurred days 84 after the last immunization and 21 days after challenge; lung tissues were collected for CFU counting. PBS and BCG groups were used as control. There were at least 5 mice per group. (**B**) The evaluation of protective efficacy against H37Ra. Eighty-four days after the last vaccination, mice were challenged with 5 × 10^6^ CFU of *M. tuberculosis* H37Ra via intranasal route. Twenty-one days later, the animals were euthanized and CFU counts in lungs were determined. Results are presented as mean ± SD of log10 CFU/lung from groups of 5–6 mice. Unpaired two-tailed Student’s *t*-tests were used to compare the two groups and one-way analysis of variance including a Tukey post hoc test was used to compare three or more groups. ** *p* < 0.01.

The original Figure S2 was thus removed from the Supplementary Material, while Figure S1 was remained.

The authors state that the scientific conclusions are unaffected. This correction was approved by the Academic Editor. The original publication has also been updated.
