# Supplementary material for: A VLP-Based Vaccine Displaying HBHA and MTP Antigens of Mycobacterium tuberculosis Induces Protective Immune Responses in M. tuberculosis H37Ra Infected Mice
Source: Vaccines (Basel). 2023 May 4;11(5):941. doi: 10.3390/vaccines11050941 (PMC10224509; doi:10.3390/vaccines11050941)
Supplement: Supplementary file 1 [file vaccines-11-00941-s001.zip › vaccines-2331684-supplementary.pdf]

Supplementary information:

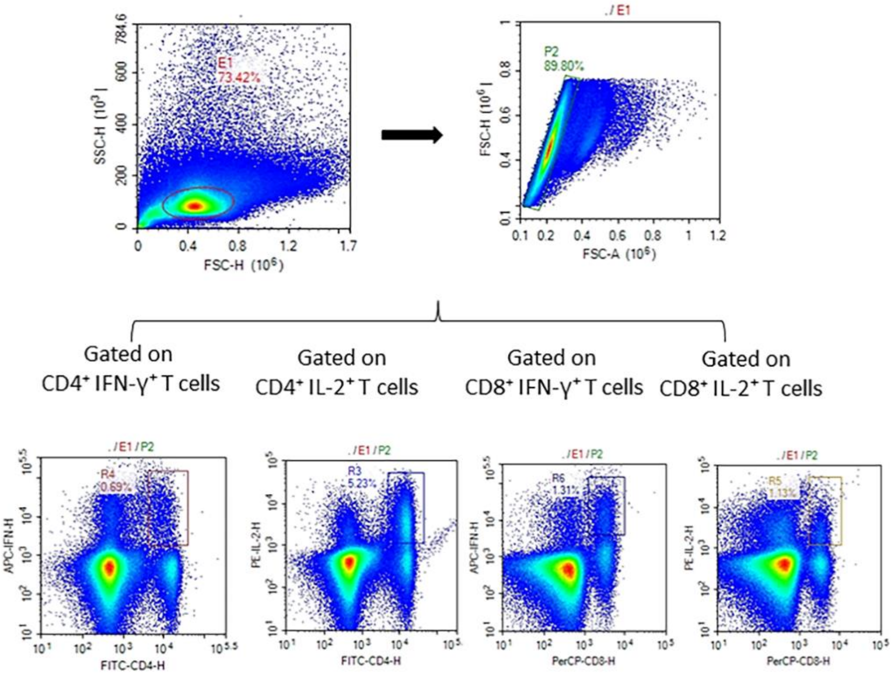

Figure S1. Flow cytometry gating strategy. The spleen lymphocytes were gated first on parameters SSC-H and FSC-H (lymphocytes), then single cells were selected by the parameters FSC-H and FSC-A (single cells). Frequencies of antigen-specific CD4 $^+$ IFN-γ $^+$ T cells, CD4 $^+$ IL-2 $^+$ T cells, CD8 $^+$ IFN-γ $^+$ T cells, CD8 $^+$ IL-2 $^+$ T cells were analyzed using flow cytometry.

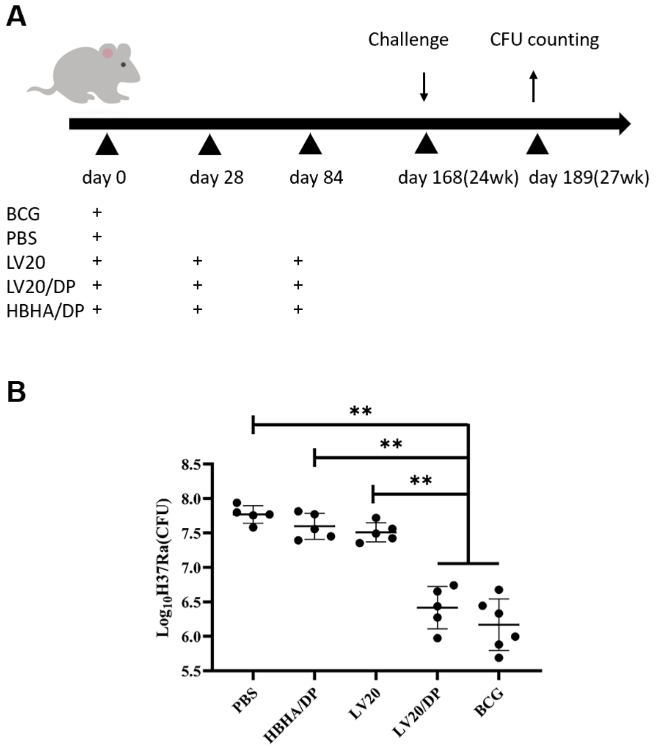

Figure S2. Immunization schedule and protective efficacy of immunization by LV20 in adjuvant DP against *M. tuberculosis* H37Ra infection. (A)The immunization schedule to assess protective efficacy.C57BL/6 mice were immunized with BCG and PBS one-time inoculation on day 0. HBHA/DP, LV20 and LV20/DP were immunized subcutaneously three times on day 0, 28 and 84. H37Ra (5×10^6 CFU in 50μl per mouse) intranasal challenge at days 84 after the last immunization and days 21 after challenge, lung tissues were collected for CFU counting. PBS and BCG groups were used as control. The number of mice per group was at least 5. (B) The evaluation of protective efficacy against H37Ra. Eighty-four days after the last vaccination, mice were challenged with 5×10^6 CFU of *M. tuberculosis* H37Ra by aerosol route. Twenty-one days later, the animals were euthanized and CFU counts in lungs were determined. Results are presented as mean ± SD of log10 CFU/lung from groups of 5-6 mice. Unpaired two-tailed Student's t-tests was used to compare two groups and one-way analysis of variance by a Tukey post hoc test to compare three or more groups. ***P* <0.01.
